# Synthesis and Evaluation of the Antimicrobial Activity of Spiro-4H-pyran Derivatives on Some Gram Positive and Gram Negative Bacteria

**Published:** 2017

**Authors:** Pardis Nazari, Aliyeh Bazi, Seyed AbdulMajid Ayatollahi, Hadi Dolati, Seyedeh Mahbobeh Mahdavi, Laleh Rafighdoost, Marzieh Amirmostofian

**Affiliations:** a *Depatment of Pharmaceutical Biotechnology, School of Pharmacy, Zabol University of Medical Sciences, Zabol, Iran. *; b *Phytochemistry Research Center, Shahid Beheshti University of Medical Sciences, Tehran, Iran.*; c *Depatment of Medicinal Chemistry, School of Pharmacy, Zabol University of Medical Sciences, Zabol, Iran.*

**Keywords:** Spiro-4H-pyran, one-pot reaction, three-component reaction, *Staphylococcus aureus*, *Streptococcus pyogenes*, Microbroth dilution, Disk diffusion

## Abstract

Infections are one of the most important causes of death, disability and inappropriate conditions for millions of people around the world. Therefore, the development in prognosis, prevention and treatment of infectious diseases made a considerable progress in designing and synthesis of new antimicrobial drugs. Nowadays, due to the increase in microbial resistance, discovery of new compounds with broad spectrum effects is granted. 4H-pyran derivatives and spiro compounds are the most important fragments in some effective drugs with antimicrobial activity. Therefore, in this study, 6 compounds with spiro-4H-pyran core were synthesized and evaluated for their antimicrobial activity against four different bacterial species using microbroth dilution and disk diffusion methods. Minimum inhibitory concentration (MIC) has been measured for each compound and also for the standard antibiotic, gentamicin, and they were all compared together. According to our result, one of the spiropyran derivative (5d) containing both the indole and the cytosine ring, has been shown good antibacterial effects against standard and clinical isolates of *Staphylococcus aureus* and *Streptococcus pyogenes*.

## Introduction

Despite a century of often successful struggles for prevention and treatment of infectious diseases, the issue remains an important worldwide problem in public health which brings about the loss of more than 13 million individuals each year (1).In the last decades, antibiotic resistance, especially in the developing countries, elevated with an accelerating steep. The noticeable factors which can cause the rise of antibiotic-resistant strains are the free access of patients to antibiotics and the use of subtherapeutic doses. In addition, the lack of an effective, forethought, and judicious antibiotic policy, and also the insufficient intervention of the medical professions to supervisory programs, if any exist, spread the antibiotic-resistance (2). Increasing transportation facilities and thus international travels, provides great opportunities for antibiotic resistant bacteria dissemination to other countries (3,4). In recent years, pharmaceutical companies spent less budget in the field of antimicrobial discovery research and development and as a result the number of new antibiotics reached approval for human use was too low. In addition, there has been less innovation in the structural and pharmaceutical characteristics of designed and synthesized antimicrobials, so the outcome is a shortage of newer, safer and more effective antimicrobial agents. The consequences of these events are the emergence and spread of antibiotic resistant bacteria which followed by the increasing incidence of potentially serious MDR bacterial infections (5-7).

**Figure 1:**
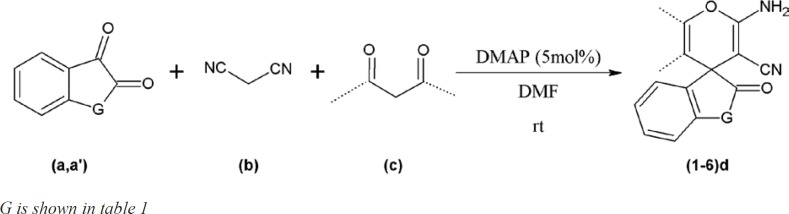
A detailed mechanism of preparation of spiroaminopyranswith DMAP

**Figure 2:**
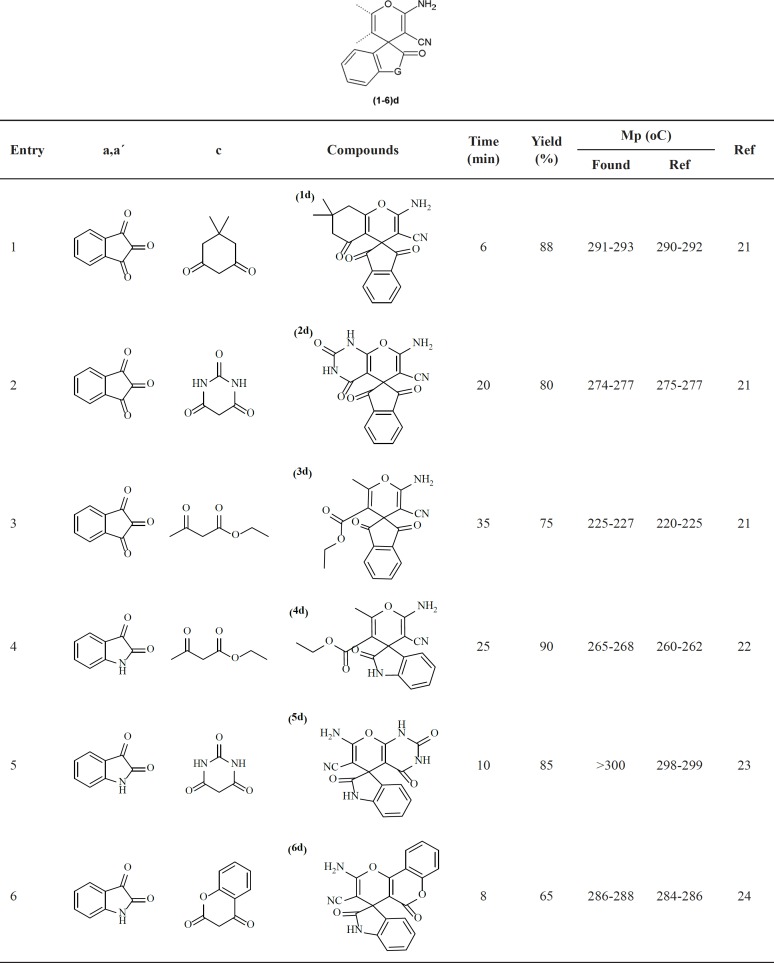
Gram staining of isolated clinical strains, a)*S.aureus*, b)*S.pyogenes*, c)*E.coli*, and d)*P.aeruginosa*.

**Scheme 1:**
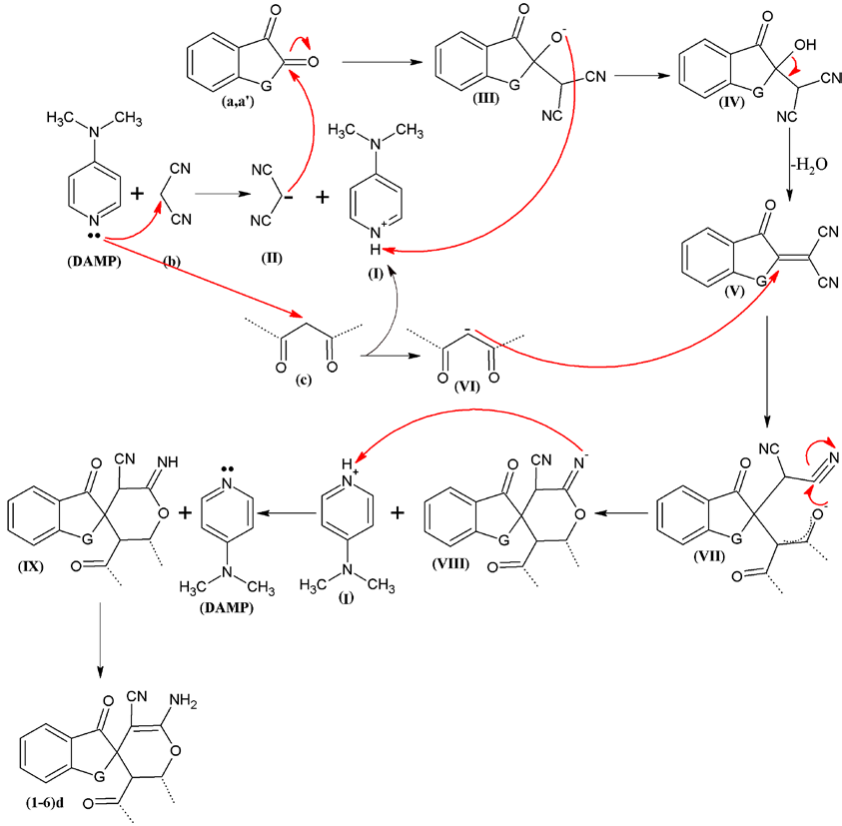
One-pot three-component synthesis of spiropyran compounds(1-6)d.

**Table 1:** The structures and yields of compounds (1-6)d

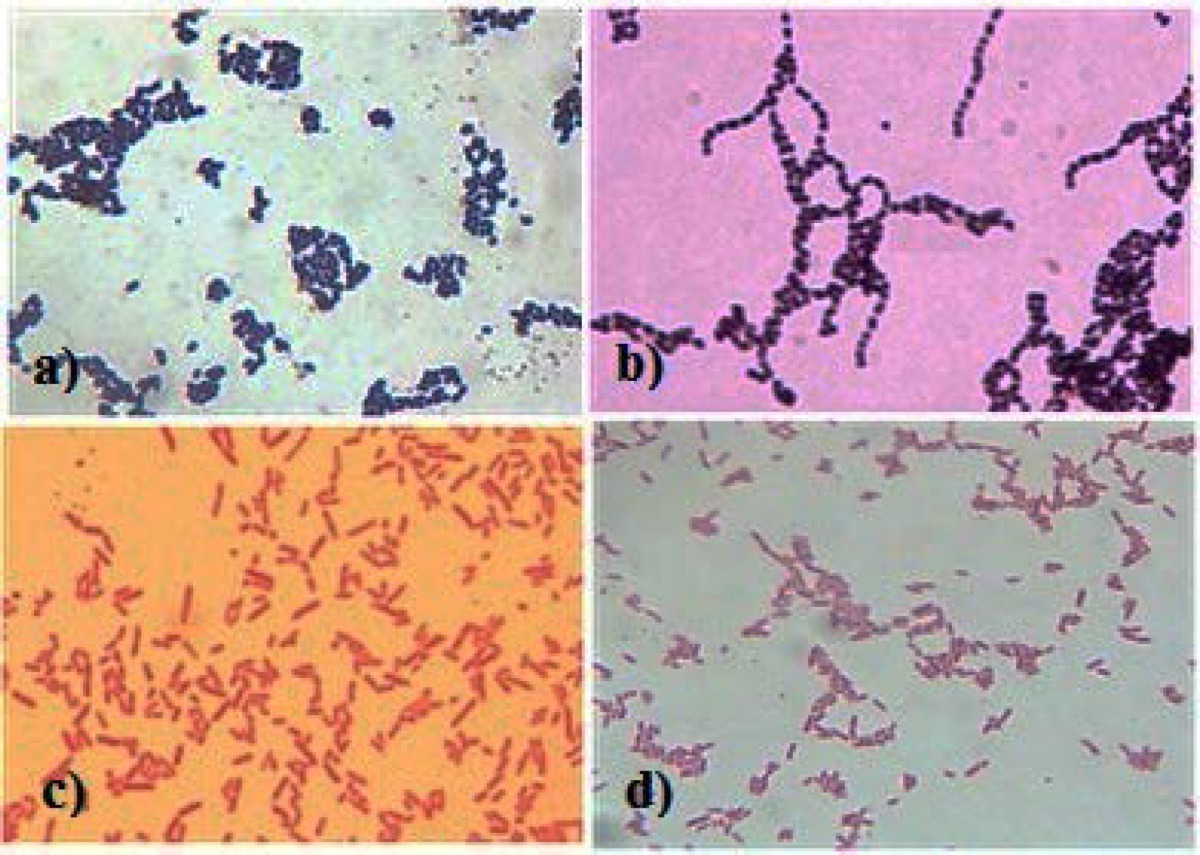

**Table 2:** Evaluation of molar ratio of DMPA and solvents in the synthesis of compound 1d

**Entry**	**Solvent**	**Catalist** ***DMPA*** **(mol %)**	**Time** **(min)**	**Yield (%)**
1	C_2_H_5_OH	(10 mol %)	15	69
2	CH_3_OH	(10 mol %)	12	65
3	DMF	(10 mol %)	5	75
4	Et_2_O	(10 mol %)	160	trace
5	THF	(10 mol %)	30	42
6	H_2_O	(10 mol %)	10	62
7	DMSO	(10 mol %)	20	60
8	CHCl_3_	(10 mol %)	30	65
9	CH_2_Cl_2_	(10 mol %)	10	57
10	n-hexan	(10 mol %)	120	72
11	DMF	(2mol %)	10	76
12	DMF	(5mol %)	6	88
13	DMF	(15mol %)	5	63
14	DMF	(20mol %)	3	58

**Table 3:** Spiroaminopyrans MIC (µg/mL) against clinical and standard isolates by microbroth dilution

**MIC (µg/mL)**								
**Clinical isolates**					**Standard strains**			
** Strains**	***S. aureus***	***S.*** ***pyogenes***	***E.*** ***coli***	***P. aeruginosa***	***S. aureus***	***S.*** ***pyogenes***	***E. coli***	***P. aeruginosa***
**Compounds **								
Gentamicin	4	8	16	16	4	8	32	16
1d	512≤	512≤	512≤	512≤	512≤	512≤	512≤	512≤
2d	512≤	512≤	512≤	512≤	512≤	512≤	512≤	512≤
3d	512≤	512≤	512≤	512≤	512≤	512≤	512≤	512≤
4d	512≤	512≤	512≤	512≤	512≤	512≤	512≤	512≤
5d	32	64	512	512	128	128	512≤	512≤
6d	512≤	512≤	512≤	512≤	512≤	512≤	512≤	512≤

**Table 4:** Inhibition zone diameter of compound 5d against S. aureus and S. pyogenes

**Inhibition zone diameter** **(mm)**		**16**	**32**	**64**	**128**	**Gentamicin disk** **(10 µg)**
*S. aureus*	clinical isolates	10.2 ± 1.44	12.6 ± 1.12	15.6 ± 1.71	19.6 ± 1.52	21.5 ± 1.45
	standard species	0	0	0	10.5 ± 1.45	25.5
*S. pyogenes*	clinical isolates	0	0	10.4 ± 0.88	12.6 ± 0.77	14.5 ± 0.22
	standard species	0	0	0	9.5 ± 0.56	17

In fact, amino-4H-pyrans and constitutes are biologically active compounds with a wide spectrum of activities such as antimicrobial, antifungal, antitumor (8), anticoagulant (9), diuretic (10), spasmolitic, and antianaphylactic activities (11). Furthermore, amino-4H-pyrans represent the building blocks of a series of considered bioactive products (5). On the other hand, spiroheterocycles have recently attracted much attention as an important class of heterocyclics which have useful biological and pharmacological properties, such as anticonvulsants (12), antimicrobials and antibacterials (13, 14), anti-tubercular agents (15), anticancer agents (16), antidermatitis agents (17), pesticides (18), antioxidants (19), and enzyme inhibitors (20).

Increase in microbial resistance worldwide against current antimicrobial agents results in the necessity of comprehensive evaluation of prepared new innovative agents. In this regard, 6 chemical compounds with spiroaminopyran nucleus (Table1) were synthesized. The synthesis was carried out by one-pot, three-component reaction of satin or ninhydrin with malononitrile and beta-dicarbonyl substances in the presence of 4-dimethylaminopyridine (DMAP) as catalyst (Scheme 1). The compounds were examined by usingmicrobroth dilution and disk diffusion methods on a selected group of gram positive and gram negative bacteria.

## Experimental

*Chemicals*

All chemicals, reagents and solvents used in this study were purchased from Merck AG, Fluka and Aldrich Chemical Company and were used without further purifications. Infrared spectra were acquired using a Perkin Elmer Model 1420 spectrometer. A BruckerAMX-300 MHz instrument (Brucker Biosciences, Germany) was used to acquire 1HNMR spectra with TMS as internal standard. Chloroform-D and DMSO-D6 were used as solvents. Coupling constants (J) values are calculated in hertz (Hz) and spin multiplicities are given as s (singlet), d (double), t (triplet), and m (multi). The mass spectra are available in refrences (21-24).

*Chemistry*

*General procedure for the preparation of compounds (1-6)d*

In a GeSneral procedure, a mixture of ninhydrina (1 mmol) or isatinaʹ (1 mmol) and malononitrileb (1 mmol) in 2 mL of DMF were stirred in a 5-mL round-bottomed flask in the presence of 5 mol% of 4-dimethylaminopyridine (DMAP) at room temperature. Then beta-dicarbonyl compounds **c **(1 mmol) was added to the mixture and the contents were stirred at room temperature for appropriate time (Scheme 1, Table 1). After the completion of the reaction as followed by TLC, water (5 mL) was added to the mixture and the solid product was collected by filtration and washed with almost 20 mL of water and the purified compounds (1-6) d were obtained (Table 1).

*2-amino-7,7-dimethyl-1ʹ,3ʹ,5-trioxo-1ʹ,3ʹ,5,6,7,8-hexahydrospiro[chromene-4,2ʹ-indene]-3-carbonitrile (1d)*: Yellow powder, IR (KBr) (λ_max_, cm^-1^): 3376, 3311, 3143, 2192, 1722, 1682, 1655.^1^H NMR (DMSO-d_6_, 300 MHz):8.04-7.97 (m, 4H, Ar-H), 7.66 (s, 2H, NH_2_), 2.60 (s, 2H, CH_2_), 2.18 (s, 2H, CH_2_), 1.02 (s, 6H, 2CH_3_).^13^C NMR (DMSO-d_6_): 199.7, 196.0, 166.5, 159.8*, *140.5, 136.6, 123.1, 116.8, 110.0, 53.0, 51.7, 48.9, 32.4, 27.1.

*7ʹ-amino-1,2ʹ,3,4ʹ-tetraoxo-1,1ʹ,2ʹ,3,3ʹ,4ʹ-hexahydrospiro[indene-2,5ʹ-pyrano[2,3-d]pyrimidine]-6ʹ-carbonitrile (* ***2d*** *)*:Yellow powder, IR (KBr) (λ_max_, cm^-1^): 3361, 3312, 3198, 2196, 1745, 1715, 1673, 1591, 1341, 1263. ^ 1^H NMR (DMSO-d_6_, 300 MHz):11.13 (s, 1H, NH), 9.33 (s, 1H, NH), 8.06–8.00 (m, 4H, Ar-H), 7.71 (s, 2H, NH_2_).^13^C- NMR (DMSO-d_6_): 200.3, 194.1, 163.3, 162.6, 160.0, 156.3, 151.5, 150.9, 141.5, 140.4, 136.7, 135.1, 123.1, 122.9, 116.7, 113.0, 84.6, 57.8, 53.1, 44.0, 22.7.

*Ethyl-2ʹ-amino-3ʹ-cyano-6ʹ-methyl-1,3-dioxo-1,3-dihydrospiro[indene-2,4ʹ-pyran]-5ʹ-carboxylate(* ***3d*** *)*: Yellow powder, IR (KBr) (λ_max_, cm^-1^): 3334, 3176, 2926, 2192, 1659, 1429, 1275, 1166, 1089, 1013.^1^H NMR (DMSO-d_6_, 300 MHz):7.99–7.84 (m, 3H, Ar-H), 7.81–7.76 (m, H, Ar-H), 6.90 (s, 2H, NH_2_), 4.31–4.15 (m, 2H), 2.25 (s, 3H), 1.35- 1.42 (m, 3H).^13^C NMR (DMSO-d_6_): 193.9, 168.7, 167.5, 164.2, 143.9, 137.4, 137.3, 133.2, 125.7, 125.2, 116.7, 107.3, 60.6, 56.7, 14.5, 14.0.

*Ethyl-2ʹ-amino-3ʹ-cyano-6ʹ-methyl-2-oxospiro[indoline-3,4ʹ-pyran]-5ʹ-carboxylate(* ***4d*** *)*:Whitepowder, IR (KBr) (λ_max_, cm^-1^): 3482, 3283, 3158, 3078, 2978, 2191, 1723, 1701, 1676, 1618.^1^H NMR(DMSO-d_6_, 300 MHz):10.40 (s, 1H, NH), 7.18 (t, 1H, J=7.6 Hz,Ar-H),7.16 (s, 2H, NH_2_), 7.05 (d, 1H, J=7.1 Hz,Ar-H), 6.94 (t, 1H, J=7.3 Hz,Ar-H), 6.79 (d, 1H, J=7.6 Hz,Ar-H), 3.70–3.79 (m, 2H, CH_2_), 2.29 (s, 3H, CH_3_), 0.74 (t, 3H, J=7.0 Hz, CH_3_).^13^C NMR (DMSO-d_6_): 178.6, 164.5, 158.9, 158.5, 142.1, 134.5, 128.5, 123.4, 121.8, 117.5, 109.3, 104.6, 60.2, 56.4, 48.9, 18.5, 13.0.

*7ʹ-amino-2,2ʹ,4ʹ-trioxo-1ʹ,2ʹ,3ʹ,4ʹ-tetrahydrospiro[indoline-3,5ʹ-pyrano[2,3-d]pyrimidine]-6ʹ-carbonitrile (* ***5d*** *)*: Whitepowder, IR (KBr) (λ_max_, cm^-1^): 3559, 3286, 3215, 2199, 1717, 1642, 1324.^1^H NMR (DMSO-d_6_, 300 MHz):12.09 (s, 1H, NH), 11.01 (s, 1H, NH), 10.45 (s, 1H, NH), 7.33 (s, 2H, NH_2_) 7.09–7.28 (m, 4H, Ar-H).^13^C NMR (DMSO-d_6_): 177.7, 161.5, 158.3, 153.8, 149.6, 142.5, 133.6, 128.3, 123.7, 121.7, 117.0, 109.2, 86.5, 57.7, 46.6.

*2ʹ-amino-2,5ʹ-dioxo-5ʹH-spiro[indoline-3,4ʹ-pyrano[3,2-c]chromene]-3ʹ-carbonitrile6d)*: Whitepowder, IR (KBr) (λ_max_, cm^-1^): 3324, 3178, 2201, 1721, 1673, 1610, 1471, 1358.^1^H NMR (DMSO-d_6_, 300 MHz):10.67 (s, 1H, NH), 7.92 (d, 1H, J=7.9 Hz,Ar-H), 7.73 (t, 1H, J=8.1 Hz,Ar-H), 7.66 (s, 2H, NH_2_), 7.50 (t, 1H, J=7.6 Hz,Ar-H), 7.46 (d, 1H, J=8.4 Hz,Ar-H), 7.19 (t, 2H, J=7.5 Hz,Ar-H), 6.90 (t, 1H, J=7.4 Hz,Ar-H), 6.83 (d, 1H, J=8.0 Hz,Ar-H).^13^C NMR (DMSO-d_6_): 177.2, 158.4, 155.0, 152.0, 142.0, 133.6, 133.0, 128.9, 125.0, 124.1, 122.6, 122.0, 116.9, 116.6, 112.4, 109.5, 101.4, 57.0, 47.5.

*Microbial strains*

From June 2014 to January 2015, five clinical isolates of each microbial strain, including *Staphylococcus aureus*, *Escherichia coli*, *Pseudomonas aeruginosa,* and *Streptococcus pyogenes*were recovered from human infections. The clinical specimens isolation was carried out based on standard bacteriological procedures. Then the isolates were identified by use of gram staining technique, differential cultures, and routine biochemical tests. Reference strains which were used in this study consist of* S. aureus *(ATCC25923),* E. coli *(ATCC8739),* P. aeruginosa *(PTCC1599),* S. pyogenes *(PTCC1447).

*Antimicrobial susceptibility testing*

A stock solution (1024 µg/mL) of each compound (1-6)d was prepared in dimethyl sulfoxide 20% to be used in antimicrobial susceptibility testing. Evaluation of spiroaminopyran compounds antibacterial activities was performed by broth microdilution and disk diffusion methods. For this purpose, 100 µL of the Mueller Hinton broth medium was added to each of the 96 wells in sterile microtiter plates. A series of doubling dilutions for each compound were made in microtiter plate wells, in the concentration range of 1 to 512 µg/mL for each of six different compounds. Fresh culture of each clinical and standard strain was harvested and microbial colonies were suspended in 0.9% NaCl solution. The microbial concentration was adjusted to 0.5 McFarland standard by turbidimetric method. Each well was inoculated with the prepared suspension in the way that the final 5×10^5^ CFU/ml bacterial concentration in the well was obtained. The plates were incubated at 37°C for 24 h. All the mentioned procedures were done also for gentamicin as the standard antimicrobial agent by the difference that a stock solution of 1,024 mg/liter gentamicin powder in sterile water was prepared. The lowest compound concentration that visibly inhibits microbial growth was reported as the minimum inhibitory concentration (MIC). It should be noted that on each plate, positive control containing broth medium with bacterial inoculum and negative control which was prepared by adding DMSO (20%) to broth medium were noticed and all the procedure was according to the standard protocol of NCCLS broth microdilution MIC determining method.

For the semi quantitative disk diffusion procedure, sterile 6 mm paper disks which were impregnated by spiroaminopyran compounds were used. Mueller Hinton agar plates were inoculated by a swab from 1.5×10^8 ^CFU/ml bacterial suspension and the antibiotic contained disks were placed on the plate surface. The amount of each compound was loaded on paper disks chosen based on microbroth dilution results in the range of 16, 32, 64, and 128 µg. The plates were incubated for 24 h at 37 °C. By the way, a 10 µg gentamicin standard disk also was included. Following incubation, the inhibition zone diameter around each disk was measured in millimeters. All the procedure was done according to the standard protocol of NCCLS disk diffusion susceptibility method (25). All the above tests for each compound were done in duplicate for three times.

## Results and Discussion

In this article, we have described an efficient one-pot, three-component reaction for the synthesis of spiroamino-pyran compounds. Several methods have been reported for the synthesis of spiropyran-fused heterocycles. Although each of the known methods for the synthesis of spiropyran systems has its advantages, still there is further scope to develop a new methodology using a less expensive catalyst under mild reaction conditions and applicable to a wide range of substrates in great demand. As mentioned, in this paper, 4-dimethylaminopyridine (DMAP) (26) was used as an efficient important and useful catalyst in this one-pot three-component reaction for the synthesis of spiroamino-pyran(1-6)d compounds.

At first, we tested the reaction of ninhydrin (a) malononitrile (b) and dimedon (c) as a simple model substrate to find the optimal conditions and amount of catalyst required for the synthesis of spiropyran derivatives in different solvent and molar ratio of DMAP as catalyst. The results are summarized in Table 2.

We started our investigation by reacting ninhydrin (a) with malononitrile (b) and dimedon (c) in the presence of (DMAP) 10 mol% in various solvents (Scheme 1-Table 2). It was found that when the reaction was carried out with Et_2_O as solvent, only trace product was detected (Table 2, entry4). As shown in Table 2, THF, H_2_O, DMSO, CHCl_3_ and CH_2_Cl_2_, gave low yield and long reaction time of product (Table 2, entries 5-9). The yield of the reaction in DMF is greater and the experiments obviously showed an increased reaction rate in DMF as compared to other solvents such as C_2_H_5_OH, CH_3_OH, n-hexan (Table 2 , entries 1,2,10). The optimized amount of the catalyst was determined using different mole percent of DMAP such as (2 moL %) (5 moL %). (15 moL %) and (20 moL%) (Table 2). Accordingly, the best yield in the shortest reaction time was obtained using the 5 moL% of DMAP (Table 2). The subsequent increase in the catalyst load did not improve the product yield of the reaction and 2 mol% of DMAP catalyzed the reaction but needs a longer reaction time.

Spiropyran (Ld) of ninhydrin, malononitrile and dimedonin in the presence of DMAP 

(5 moL%) and using DMF as a solvent at ambient temperature was obtained in 88 % yield. The scope and efficiency of this approach was explored for the synthesis of a variety of spiropyrans by isatin or ninhydrin with malononitrile and beta-dicarbonyl compounds and results are summarized in Table 1. Fortunately, it was found that nearly all of the corresponding products were obtained in good yields.

A detailed proposed mechanism of the reaction is shown in Figure 1. As shown in Figure 1, using the DAMP as a catalyst, the knoevenagel reaction is initiated by formation of malononitrile anion (II). In this mechanism, the anion (II) is attacked to ninhydrin (a**)** orisatin** (**a**ʹ)** to yield the compound III which undergoes the knoevenagel condensation to afford the compound **V** by elimination a molecule of water. The beta-dicarbonyl compounds (c) activated by DAMP (VI), is attacked to the compound V. Also, the new intermediate compound VII undergoes the other spontaneously reactions to obtain the final products the spiroaminopyran compounds **(**1-6) d.

The antimicrobial activity of the spiroamino-4H-pyran compounds (1-6) d was evaluated against a standard and 5 isolated clinical bacterial strains of *Staphylococcus aureus*, *Streptococcus pyogenes*, *Escherichia coli,* and *Pseudomonas aeruginosa*, which were identified by use of gram staining (Figure 2) and biochemical tests, by the disk diffusion and broth microdilution methods (25). Preliminary screening and MIC determination of the spiroaminopyran derivatives was performed in different concentrations encompassed the range of 1, 2, 4, 8, 256 and 512 µg/mL.

The MIC (µg/mL) of mentioned compounds (1-6) dagainst tested strains are presented in Table 3. Amongst all of these compounds, the cytosine derivative of spiroaminopyran, compound 5d, was the most effective one with the most activity against the clinical isolates of both *Staphylococccus aureus* and *Streptococcus pyogenes*. Compound 5d, especially, exhibited good activity against clinical isolates of *S. aureus* and *S. pyogenes* with MICs of 32 and 64 µg/mL, respectively (Table 3). The compound showed moderate antibacterial activity against the standard *S. aureus* and *S. pyogenes*. It was inactive against standard and clinical isolates of gram negative bacteria (MIC≥512) (Table 3).

Growth inhibition was recorded by measuring the inhibition zone diameter at the end of 18-24 h. incubation and the results were shown in Table 4. Gentamicin was used as standard drug in this assay.

Since other derivatives of spiroaminopyran than no 5 ddidnot show any significant effects on investigated bacteria, the results of inhibition zone diameter measurements were not included.

By comparing the inhibiting effects of the spiroaminopyran derivatives (1-6)d we can deduce that the presence of indol ring and cytosin-like moiety conferred the highest antibacterial activity in these substances. In some researches, the compounds containing only cyanide and amino substituents on aminopyran derivatives have not shown appropriate effects against bacteria (27-29) which is also shown in our investigation. Compounds with just spiro-pyran and indole-pyran core showed a potent antibacterial activity (28, 30) and some others, containing spiroaminopyran-indole moiety exhibited very high antimicrobial effects (28, 31, 32). Also in some studies, compounds with cytosin like moiety has good antibacterial activities (33, 34).

It is fascinating to notice that between all spiropyran compounds (1-6) d in this study, compound 5d with spiro[aminopyran-indole] containing cytosine-like ring in its structure, has significant antimicrobial activities which is in accordance with previous published surveys.

## Conclusion

In the present study, a series of spiro-aminopyran derivatives were synthesized as a novel and efficient catalytic method through a one-pot, three-component reaction catalyzed by DMAP. Mainly, the significant advantages of this protocol are the excellent product yields, broader substrate scope, short reaction time, high reaction rates, easy work-up (no chromatographic separation) and benign reaction conditions (room temperature and one-pot manner).

They were evaluated for their antimicrobial activities against some gram positive and gram negative bacteria. The results showed that compound 5d has the greatest inhibitory activity against tested microorganisms, whereas the rest of the compounds haveno considerable effects on microbial growth. Also, the most susceptible species were*S. aureus*. However, these compounds, aside from compound 5d, did not show a remarkable growth inhibitory activity against the gram negative bacteria. Whereas, based on the gentamicin results, it should be mentioned that the new compounds are so less potent in comparison with regular antimicrobial agents. It could be concluded that spiro[aminopyrans-indole] core with cytosine-like moiety, based on their antimicrobial activity, can be the next antibiotics generation.
